# Daily Travel and Wellbeing among the Elderly

**DOI:** 10.3390/ijerph17072342

**Published:** 2020-03-30

**Authors:** Margareta Friman, Lars E. Olsson

**Affiliations:** CTF Service Research Center and Department of Social and Psychological Studies, Karlstad University, SE-65188 Karlstad, Sweden; lars.e.olsson@kau.se

The purpose of this Special Issue is to introduce and demonstrate the importance of daily travel in elderly people’s lives. In doing so, we wish to present recent advances made in this emerging field. Our overall goal is to provide a broad understanding of the links between psychological wellbeing and travel, the importance of daily travel, and the different evaluations and measures used to assess the experience of daily travel and the quality of life of the elderly.

There are several different reasons for focusing on daily travel among the elderly: this population is growing; older people have similar needs to younger people, but they also have different needs when it comes to travel and activity participation; they are more active than previous generations, even those in older age brackets; and society has other expectations of the elderly today than previously in terms of, for example, their capacity to work and take care of their own needs in very old age as well. In this Special Issue, it is argued that developing a transport system that facilitates the elderly’s travel needs is not only advantageous for the elderly but also for others, i.e., more or less vulnerable groups in society. As new evidence from empirical research emerges, the nature of the relationship between daily travel and the elderly’s wellbeing becomes clearer. However, there is much still to learn, and the breadth of knowledge being introduced and discussed in this Special Issue is a step in that direction. Understanding the relationship between daily travel and wellbeing among the elderly will provide insights into ways of improving existing transport services and policies. This Special Issue consists of seven different papers covering different angles of daily travel among the elderly.

It is inevitable that the elderly, at some point and for various reasons, will need to give up driving. For many elderly people, cars are an ideal mode of transport as they are accessible, flexible, fulfill psychosocial needs, and can be used purely for pleasure without any specific goal or destination in mind. Musselwhite and Scott [1] focus on how giving up driving affects older people in terms of their health and wellbeing. In interviews with the elderly, the authors address the barriers to and enablers of life without a car. The data are analyzed and categorized according to Bourdieu’s theory of different capitals, which are defined as resources for health and wellbeing. In doing so, this paper offers a conceptual framework built on capitals that can be used to understand how to support people when they give up driving.

In their paper on modal choice, Jane Ryan [2] treats choice as an element of wellbeing. Ryan states that modal choice is important for wellbeing in terms of facilitating the fulfilment of travel needs and activity participation, with a lack thereof potentially having the opposite effect. Based on this assumption, Ryan argues that it is important to analyze the process of choice and to distinguish between actual choice and limited or no choice. Ryan asks what factors lie behind the mode choices of older people living in Sweden’s large metropolitan regions. As a result, Ryan is able to show that there are differences in how older people in Sweden perceive their options and in how they evaluate, select, and operationalize their capabilities. Ryan holds an interesting discussion about adaptation versus wellbeing in cases where older people have few modal choices, or only one. The fact that most elderly people are satisfied with very few alternatives is a good thing, but this can be a risk later in life when the chosen alternative is no longer available.

Curl, Fitt, and Tomintz [3] note that walking entails many positive, but also negative, consequences for the wellbeing of older people. The probability of falling increases as people get older, especially so when walking outdoors. Fall injuries often lead to limited mobility and physical pain, but also to decreased social contact as a result of being unable to attend social gatherings and activities. The built environment, in this paper, is acknowledged to be closely associated with the mobility of old people. Curl, Fitt, and Tomintz [3] show that it is not only actual fall injuries that limit the mobility of the elderly, but also that the perceived level of accessibility is negatively related to their fear of falling. Fear of falling, thus, limits the ability of the elderly to live the lives they want to.

A theoretical model of the relationship between perceived accessibility, satisfaction with travel, and life satisfaction is presented by Lättman, Olsson Friman and Fujii [4]. These researchers argue that older people’s perceived possibilities of accessing activities are closely linked to their wellbeing. Lättman et al. [4] define perceived accessibility in terms of perceived possibilities of accessing activities that are of importance to everyday life, or in terms of “how easy it is to live a satisfactory life considering how people travel”. Empirical testing of the model showed that perceived accessibility influences travel satisfaction, in that a higher degree of perceived accessibility (ease of travel, possibilities of traveling, and access to preferred activities) increases satisfaction with travel, while a lower degree of perceived accessibility decreases satisfaction with travel. It is concluded that different levels of perceived accessibility are significant to both travel satisfaction and older people’s satisfaction with life.

Dale Nordbakke [5] concentrates on the link between wellbeing, out-of-home activity participation, and mobility in old age. Nordbakke [5] shows that older people’s preferences, individual resources, and constraints are linked to their level of participation in out-of-home activities. These results underline the importance of not considering old people to be a homogenous group; they make different choices even when they have the same opportunities for mobility. Nordbakke [5] discusses the possibility of focusing on the concept of lifestyle in future studies in order to better capture the differences in mobility between older people.

Verma and Taegen [6] present a case regarding older people’s ability to maintain their quality of life in regions/municipalities with shrinking populations. This case illustrates an increasingly common situation in Finland, where second-home owners are growing in number. The consequence to the municipality is that a proportion of its residents, who need access to services during certain periods of time, do not pay taxes to that specific municipality. Thus, the tax base does not allow new residential areas to be built for elderly people wishing to live centrally, within walking distance of stores and services, a desire that often arises in relation to giving up driving. Based on this analysis, the authors discuss how small and shrinking municipalities can act in order to make it easier for permanent residents who want to stay within the municipality, while simultaneously remaining attractive to second-home owners who are important as regards livability. Innovative transport solutions adapted to older people, new and innovative services, and different types of meeting places in city centers are all suggestions for further development.

Bergerfurt et al. [7] look into how loneliness and life satisfaction can be explained in terms of public space use and mobility patterns. By analyzing data from the residents of three neighborhoods of a Dutch city, the effects on public space use, loneliness, and life satisfaction that are due to personal characteristics, mobility, and social neighborhood characteristics are examined. Based on these findings, it is argued that loneliness has a negative effect on life satisfaction, but that the effect of public space use on loneliness and life satisfaction is limited. This analysis supports a recommendation to focus on creating neighborhoods that are highly walkable and accessible, where green spaces and public transport facilities are present, and to promote physical activity among all residents.

In this Special Issue, seven papers have been published that focus on daily travel and wellbeing among the elderly. These seven papers present different perspectives and issues that are important to the further development of daily transport adapted to an aging population. As several of these papers show, an aging population does not need to be described as a problem that is costly and problematic; rather, an aging population may actually be a good thing—both for all of us and for the planet. These seven papers present new opportunities for the development of future travel opportunities. The research presented shows how important it is to include aspects such as accessibility, health, heterogeneity, and individual resources when developing new transport solutions that suit not just the elderly but also everyone else in the community. Increasing accessibility to the outdoor environment, to travel opportunities, and to everyday services will provide municipalities and regions with the prospects to change and even reduce resident car use. The overall aim of this Special Issue is to provide ideas and directions regarding future research. In light of these seven papers, we have concluded that there is a need for knowledge on how to support active lifestyles for the elderly and their decision to give up driving. There is also a need for research on how to prevent loss of wellbeing of the elderly, or to support gains in this, when using alternative travel modes. The overall goal of every transport policy should be to provide sustainable and accessible travel to the elderly, accompanied by sustained or increased wellbeing.

During the finalization of this editorial for the Special Issue, the world has changed rapidly. Covid-19 has spread around the world at a pace that has been unprecedented, and the elderly, in particular, are at risk of severe illness. This has led to enforced isolation and exclusion from the daily life that we are used to. In this Special Issue, it has been highlighted that perceived accessibility to daily activities is important for life satisfaction, and that physical activity plays a major role in the lives of the elderly. In the near future, research that monitors and analyzes both the short- and long-term effects of restricted mobility on the wellbeing of the elderly, resulting from the Covid-19 pandemic, will provide important insights regarding future policy.
